# ITGAL infers adverse prognosis and correlates with immunity in acute myeloid leukemia

**DOI:** 10.1186/s12935-022-02684-x

**Published:** 2022-08-23

**Authors:** Ran Li, Xiaolu Wu, Kai Xue, Junmin Li

**Affiliations:** 1 Shanghai Institute of Hematology, State Key Laboratory of Medical Genomics, National Research Center for Translational Medicine at Shanghai, Ruijin Hospital, Shanghai Jiao Tong University School of Medicine, Shanghai, China; 2 Department of Child Health Care, Women’s Hospital of Nanjing Medical University (Nanjing Maternity and Child Health Care Hospital), Nanjing, China

**Keywords:** Acute myeloid leukemia, ITGAL, Prognosis, Myeloid‐derived suppressor cells, Cytokines

## Abstract

Integrin subunit alpha L (ITGAL) was found aberrantly expressed in multiple cancer types, suggesting its essential role in tumorigenesis. Hence, we aimed to explore its definite role in acute myeloid leukemia and emphasize its associations with immunity. Here, we found ITGAL was highly expressed in AML patients and elevated expression was associated with poor prognosis. ITGAL was associated with age and cytogenetic risk classifications, but not relevant to AML driver gene mutations. Univariate and multivariate Cox regression analyses determined ITGAL as an independent prognostic factor. The nomogram integrating ITGAL and clinicopathologic variables was constructed to predict 1-, 3- and 5-year overall survival (OS). Functional analyses revealed that ITGAL was mainly responsible for the production and metabolic process of cytokine. As for immunity, ITGAL was positively associated with MDSCs including iDCs, and macrophages in the TCGA-LAML cohort. We also found that ITGAL was positively associated with most immune checkpoint genes and cytokines. In addition, we found that ITGAL knockdown caused substantial inhibition of cell growth and significant induction of early apoptosis in AML cells. The xenograft study indicated that ITGAL knockdown prolonged the survival of recipient mice. Overall, ITGAL is an independent prognostic factor and is closely related to the number of MDSCs and cytokine production.

**Supplementary Information:**

The online version contains supplementary material available at 10.1186/s12935-022-02684-x.

## Introduction

Acute myeloid leukemia (AML), a malignant clonal tumor of the hematopoietic system, is the most common type of acute leukemia in adults [1]. Despite the continuous development of chemotherapy and the emergence of many new therapeutic targets, the five-year survival rate of AML is still around 30%, mainly due to the high relapse/refractory rate [2, 3]. Improving the first complete remission (CR) rate and overcoming relapse becomes a challenge in the treatment of AML. Therefore, new biomarkers need to be sought to better understand the molecular mechanism of AML, which also contribute to both the diagnosis and treatment of AML.

Integrins are heterodimeric integral membrane proteins composed of alpha and beta chains, playing a crucial role in leukocyte homing and cell differentiation in inflammation and cancer [4, 5]. ITGAL (Integrin Subunit Alpha L) is the gene encoding CD11a, which is located on chromosome 16p11.2, and is important in inflammatory and immune responses [6, 7]. The upregulated expression of ITGAL correlated with the risk of inflammatory bowel disease (IBD) and increased levels of proinflammatory cell surface markers in response to stimulus [8]. The emergence of efalizumab, a monoclonal antibody targeting αL, supported the evidence of the relevance between ITGAL and IBD, although it has been withdrawn from the market several years later due to the risk of progressive multifocal leukoencephalopathy [9]. In addition, CC042 mice with a loss-of-function mutation in the Itgal gene suffered from a rapidly progressive disease and failed to produce the protective cytokine gamma interferon (IFN-) in the lung upon Mycobacterium tuberculosis infection [10].

Recently, ITGAL has attracted attention in the field of oncology. ITGAL was found enriched in glioma-associated microglia (GAM) from both murine and human low-grade glioma (LGG) through RNA-sequencing. ITGAL could control microglia migration and mitogen production by increasing Cx3cr1 and Ccl5 expression and promoting microglia infiltration and proliferation [11]. Besides, bioinformatics has predicted that ITGAL can be a biomarker and prognostic indicator of prostate adenocarcinoma [12], thyroid cancer [13], melanomas [14]. However, the role of ITGAL in AML remains unknown.

In this study, we hypothesized that ITGAL can be a potential prognostic biomarker for AML patients. We performed a series of bioinformatics analyses based on the TCGA-LAML cohort to uncover the clinical implications and biological function of ITGAL in AML. We analyzed the expression level, relationships of clinical indicators, the independency of prognostic predictor, and the predictive efficacy of the integrated nomogram of ITGAL in AML to prove that ITGAL is a prognostic biomarker for AML patients. The interaction between AML cells and the immune system shapes an immunosuppressive microenvironment in AML pathogenesis [15]. ITGAL are involved in leukocyte adhesion and transmigration of leukocytes including T-cells and neutrophils, indicating the essential role of ITGAL in the immune system [4]. Hence, we assume that ITGAL may play its pro-oncogenic role via regulating the immune microenvironment in AML.

## Materials and methods

### Data acquisition

RNA-seq data (transcripts per million reads) was downloaded from UCSC Xena (https://xenabrowser.net/datapages/) based on TCGA and GTEx databases. RNA-seq data were normalized according to the description from the UCSC Xena. Clinical information was obtained from the TCGA database (Additional file 1: Table S1).

### Evaluation of ITGAL gene in AML

ITGAL expression was analyzed by Wilcoxon rank-sum test. ROC analysis was performed using the pROC R package to assess the distinguishability of ITGAL for healthy people and AML patients. The Kaplan–Meier analysis was performed using “survminer” and “survival” packages. The median expression of ITGAL was defined as the cut-off value.

### Establishment of a nomogram

To individualize the predicting value of OS in AML patients, a nomogram was made using the RMS R package based on independent clinical factors determined by Cox regression analysis. Calibration curves were used to evaluate the accuracy of this nomogram.

### Functional enrichment analysis

The limma R package was used to identify DEGs with thresholds for |logFC|> 1 and p.adj < 0.05 between high-ITGAL and low- ITGAL groups (the cut-off value is the median of ITGAL expression). Gene ontology (GO) and Kyoto Encyclopedia of Genes and Genomes (KEGG) pathway analysis were performed using the ClusterProfiler R package.

### GSEA analysis

R package ClusterProfiler was used for GSEA, which reveals the enrichment of functional gene sets between high- and low-expression of ITGAL groups. We specified adjusted P-value < 0.05 and FDR q-value < 0.25 to be statistically significant.

### Network analysis

A network is a useful way of presenting protein–protein interactions (PPI). The Search Tool for the Retrieval of Interacting Genes (STRING) database was used for the prediction of the PPI network of the top 300 DEGs, which provided us with information on the interaction relationships between proteins. The interaction score threshold of 0.4 is set as the cut-off criterion. A PPI network was drawn using Cytoscape software (NIH, National Resource for Network Biology). CytoHubba in Cytoscape software provides a user-friendly interface to explore important nodes in biological networks. The important nodes represent the hub genes [16]. In this study, the important nodes were determined by the number of interactions with genes in the network.

### Immune infiltration analysis

The GSVA package was used to perform the immune infiltration analysis between ITGAL and a total of 24 types of immune infiltrating cells. Spearman correlation analysis was used to explore the association between ITGAL and the enrichment scores of immune cells; Correlation > 0.4 and p < 0.05 is considered statistically significant. Wilcoxon rank-sum was used to analyze the enrichment scores between high- and low-expressions of ITGAL groups.

### Association of ITGAL with immune-related factors

Co-expression analysis of ITGAL with immune checkpoint genes and cytokines was conducted through Spearman analysis. TMB scores were acquired through Strawberry Perl and corrected by dividing by the total length of exons. MSI scores were determined based on somatic mutation data. Spearman’s method was used to evaluate the association of ITGAL with TMB and MSI.

### Cell culture

MOLM13 and NOMO1 cells were obtained from DSMZ and kept in RPMI-1640 (Gibco, United States) with 10% fetal bovine serum (Gibco, United States), and 1% penicillin–streptomycin (Invitrogen, United States). Human bone marrow mononuclear cells (MNCs) were obtained from LONZA and kept in IMDM (Gibco, United States) supplemented with 20% fetal bovine serum (Gibco, United States), 10 ng/ml human cytokines including SCF, TPO, FLT3L, IL-3 and IL-6 (PeproTech).

### Xenograft studies

The xenograft mouse model was established by injecting 5 × 10^6^ MOLM13 cells expressing sh-NC or sh-ITGAL into NOD mice. The human CD45^+^ cells in BM were detected by flow cytometry. Animal experiments were approved by the Ethics Committee of Ruijin Hospital Clinical Research Center Shanghai Jiao Tong University, School of Medicine.

### Quantitative real-time PCR

Total RNA from cell lines was extracted using TRIzol reagent (Invitrogen, United States). Quantitative real-time PCR (qRT-PCR) was performed as previously described [17]. The primers used for qRT-PCR:

5′-TGCTTATCATCATCACGGATGG-3′ (Forward Primer),

5′-CTCTCCTTGGTCTGAAAATGCT-3′ (Reverse Primer).

### Western blot

Western blot was performed as previously described [17]. The primary antibodies (ITGAL and β-actin) were purchased from Abcam, United States. The anti-mouse or anti-rabbit secondary antibodies were obtained from Cell Signaling Technology, United States.

### Transfection

The lentivirus containing ITGAL knockdown or a negative control sequence (NC) was obtained from OBIO (Obio TechnologyCorp, China). Transduction was performed in MOLM13 cells. Pools of stable transductants were generated by selection using puromycin (1 μg/ml) for 2 weeks.

### Colony-forming assay

Cells were transduced with lentivirus and then at the density of 1000 cells/well seeded into MethoCult H4434 Classic medium (StemCell Technologies). After 10 days, cells were counted.

### Cell proliferation/growth and apoptosis assays

The cell growth was assessed by Cell Counting Kit-8 (CCK-8) proliferation assay (Dojindo, Japan) following the manufacturer’s instructions. For apoptosis assays, Annexin V-FITC/PI cell apoptosis kit (Cat. No: KGA108, KeyGEN BioTECH) was used following the manufacturer’s instructions.

### Statistical analysis

The analysis and presentation of data and graphs were performed with R software (4.1.1). In all experiments, P-value < 0.05 was considered statistically significant. All experimental measurements are shown as the means ± SD from three independent experiments.

## Results

### ITGAL was highly expressed in AML patients

**Fig. 1:**
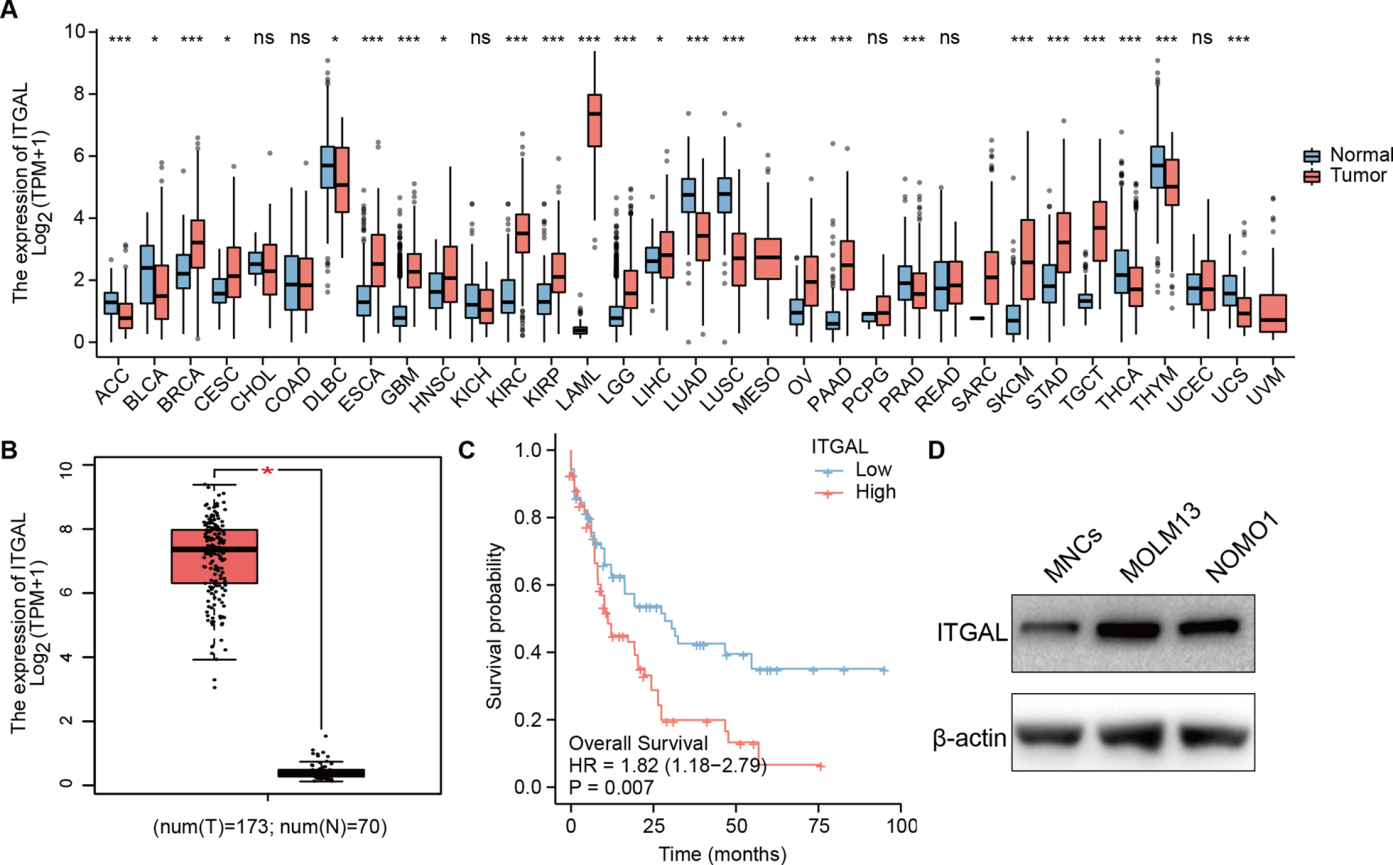
Higher expression of ITGAL was observed in AML patients. **A** Pan-cancer analysis for the expression of ITGAL based on TCGA and GTEx databases. TCGA-LAML is an AML patient cohort. A box diagram was used to display these results. The horizontal in the middle of the box represents the median, and the upper and lower sides of the box represent the upper quartile and the lower quartile. **B** The expression of ITGAL between normal controls and AML patients. **C** Kaplan–Meier survival curves for high- and low-ITGAL groups. **D** WB detection of ITGAL in MNC and AML cells

A pan-cancer analysis indicated that ITGAL was differentially expressed between normal and tumor tissues in 24 out of 33 tumor types, with AML showing the most prominent difference (Fig. 1A). In AML patients, ITGAL expression was extremely increased in tumor tissues (Fig. 1B). Kaplan–Meier curves showed that patients with high ITGAL expression (median of ITGAL expression as the cut-off value) had lower overall survival (OS) than those with low expression (Fig. 1C). Then, we used tertiles or quartiles of ITGAL expression as the cut-off value, and the results were consistent (Additional file 1: Fig. S1). The median of ITGAL expression as the cut-off value was applied for all the further analyses. ITGAL was significantly increased in AML cell lines compared with MNCs (Fig. 1D).

### Association between ITGAL expression and AML clinicopathological characteristics

**Fig. 2:**
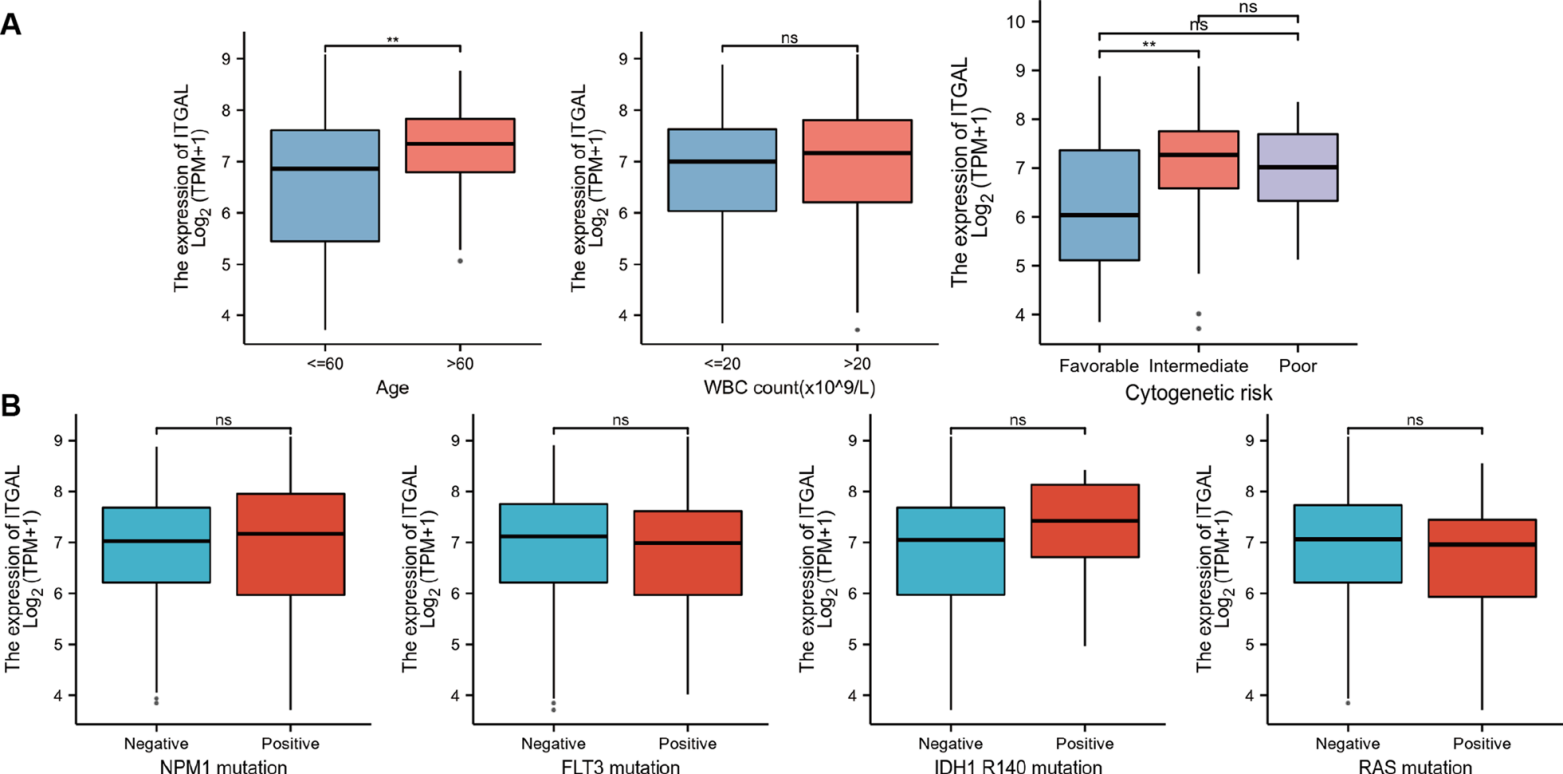
Association between ITGAL expression and clinical features. Association between ITGAL expression and age, cytogenetic risk, WBC count (**A**), and driver gene mutations (**B**)

Next, we asked whether ITGAL expression was associated with clinicopathological characteristics of AML patients. We found that ITGAL was highly expressed in age > 60 years and intermediate/poor cytogenetic risk groups. There were no significant associations with WBC count factors (Fig. 2A). In addition, ITGAL was not relevant to AML driver gene mutations (Fig. 2B).

### Construction of a nomogram model

Univariate and multivariate Cox analyses were conducted to find dependent prognostic factors. The results showed that age, cytogenetic risk, and ITGAL were dependent prognostic factors (Additional file 1: Table S2). To better establish a predictive tool for quantitative analysis of overall survival in AML patients, we constructed a nomogram with the combination of age, cytogenetic risk, and ITGAL factors (Additional file 1: Fig. S2A). The observed lines and the ideal line were closed in the calibration curve, suggesting the nomogram was accurate and reliable (Figure S2B). In addition, the AUC value of 1-, 3-, and 5-year OS was 0.769, 0.806, and 0.909 respectively, indicating the nomogram was reliable (Additional file 1: Fig. S2C).

### Functional enrichment analysis of DEGs from high-ITGAL and low-ITGAL groups

**Fig. 3:**
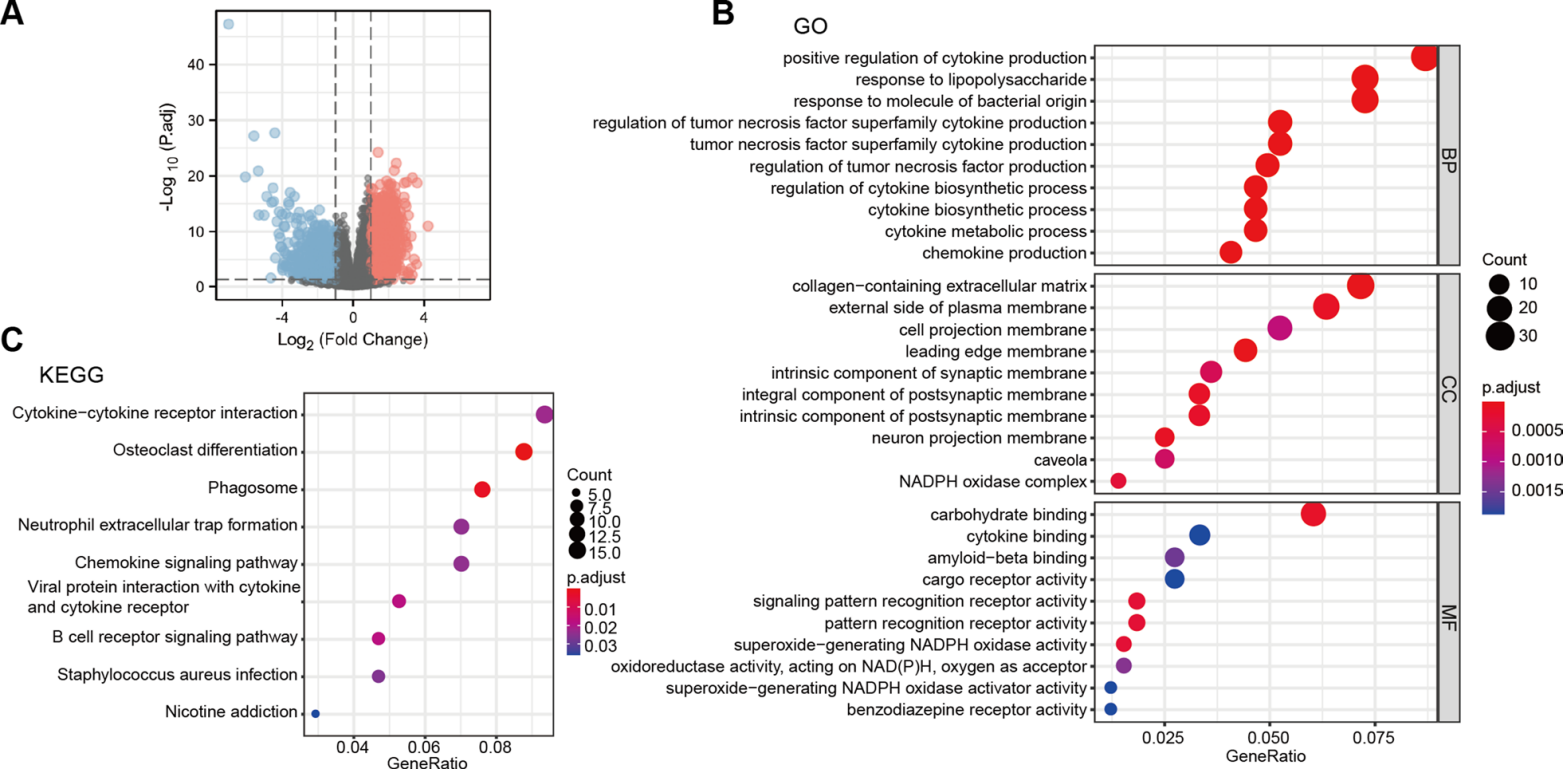
Functional enrichment analysis. **A** Volcano plot of DEGs. GO (**B**) and KEGG (**C**) analyses for DEGs

There were 2582 DEGs between high-ITGAL and low-ITGAL groups, including 1275 upregulated genes and 1307 downregulated genes (Fig. 3A). GO and KEGG analyses showed that multiple gene sets were associated with the function of ITGAL in AML. Biological process (BP) included positive regulation of cytokine production, tumor necrosis factor superfamily cytokine production, and chemokine production; cellular components (CC) included integral components of postsynaptic membrane, leading-edge membrane, and external side of plasma membrane; molecular function (MF) included amyloid–beta binding, pattern recognition receptor activity, and signaling pattern recognition receptor activity (Fig. 3B). KEGG included cytokine–cytokine receptor interaction, osteoclast differentiation, and phagosome (Fig. 3C).

### Protein–protein interaction (PPI) analysis

**Fig. 4:**
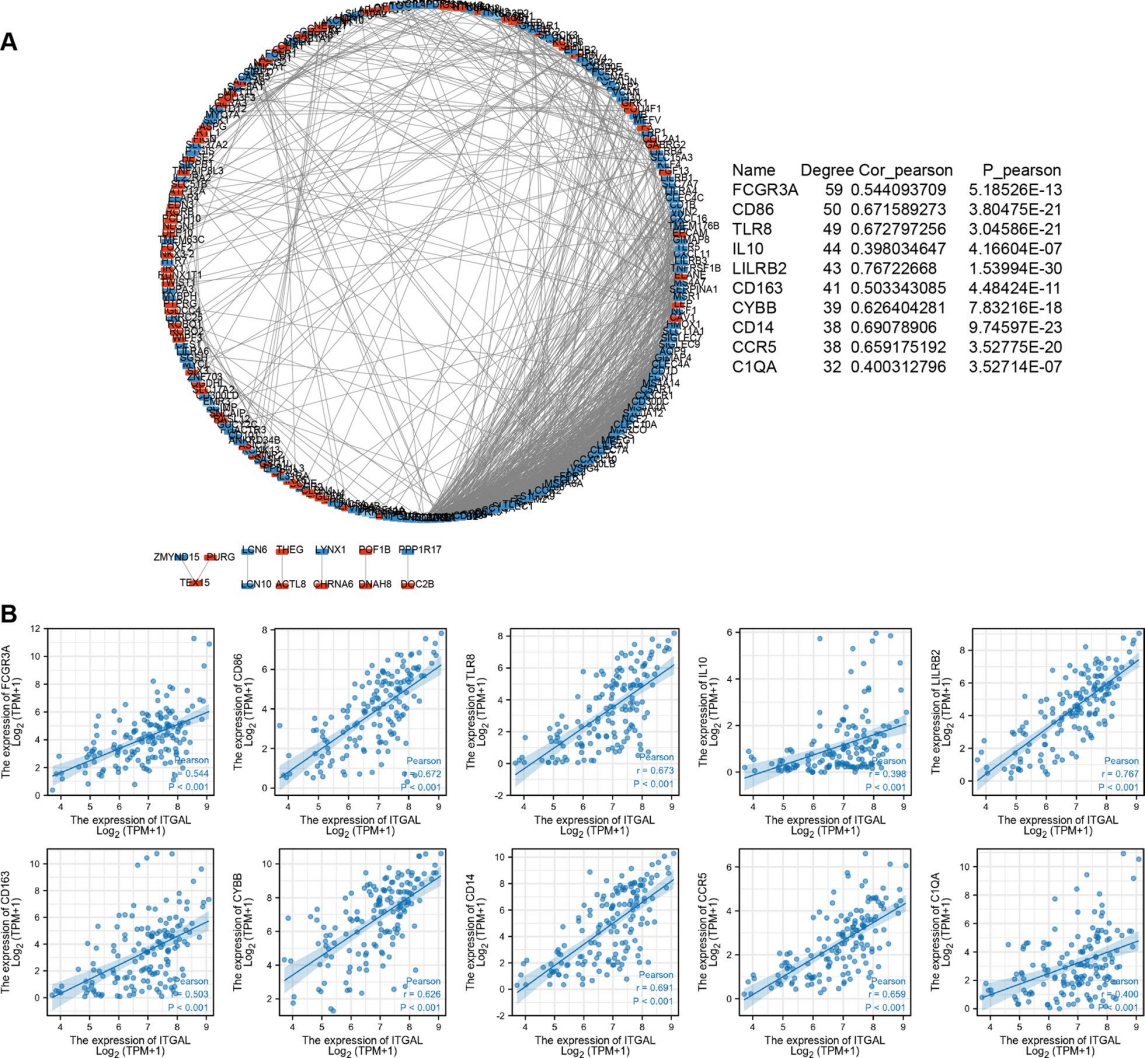
PPI analysis. **A** The PPI network of DEGs was constructed. The red rectangle represents up-regulated genes; the blue rectangle represents down-regulated genes. **B** The scatter diagram for associations between ITGAL and hub genes

To further determine the interactions of DEGs, we performed a PPI analysis of the top 300 DEGs. We constructed a network including 241 nodes and 977 edges (Fig. 4A). Then, the cytoHubba plugin was used to identify hub genes in this network and we obtained 10 hub genes, including FCGR3A, CD86, TLR8, IL-10, LILB2, CD163, CYBB, CD14, CCR5, and C1QA. We also found these hub genes were all positively associated with ITGAL expression (Fig. 4B).

### Investigation of immune infiltration

**Fig. 5:**
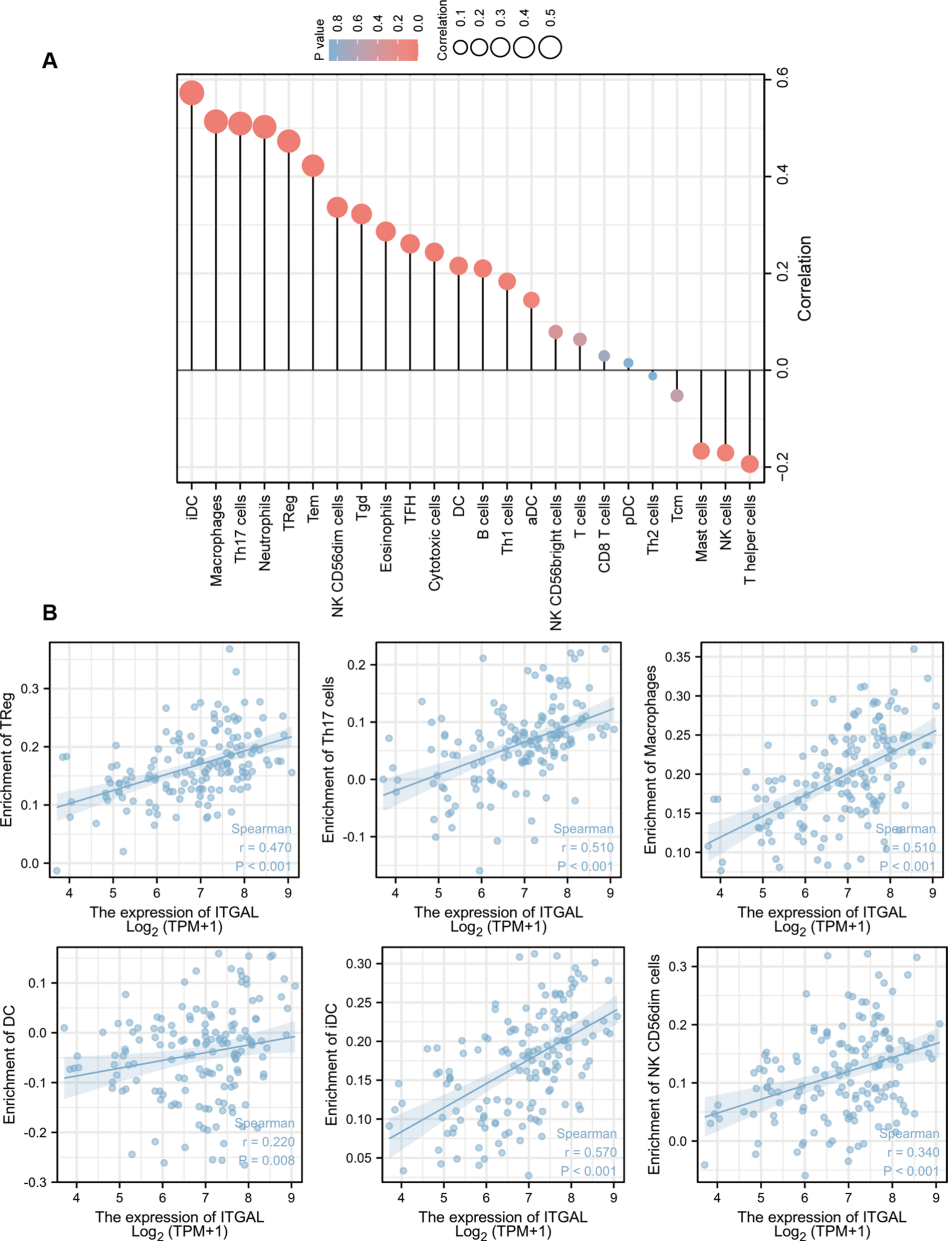
Association between ITGAL expression and immune cell infiltration in AML bone marrow microenvironment. **A** Relationships between ITGAL expression and 24 immune cell types. The size of the circle and the height of the stick represent the degree of correlation, and the depth of the color represents the size of the p-value. **B** The relationship between ITGAL and enrichment scores of Treg, Th17, macrophages, DC, iDC, and NK CD56dim cells. Enrichment scores were calculated using the “Estimate” package in R software based on the ssGSEA (single sample GSEA) concept

There is accumulating evidence that the bone marrow immune environment of AML patients is profoundly altered, contributing to the severity of the disease [18]. To investigate the relationships between ITGAL and immune cell infiltration, a correlation analysis including a total of 24 infiltrating immune cell types was conducted (Fig. 5A). We found Treg, Th17, macrophages, DC, iDC, and NK CD56dim cells were all positively associated with ITGAL expression (Fig. 5B).

### Relationships of ITGAL with immune checkpoint genes and cytokines

**Fig. 6:**
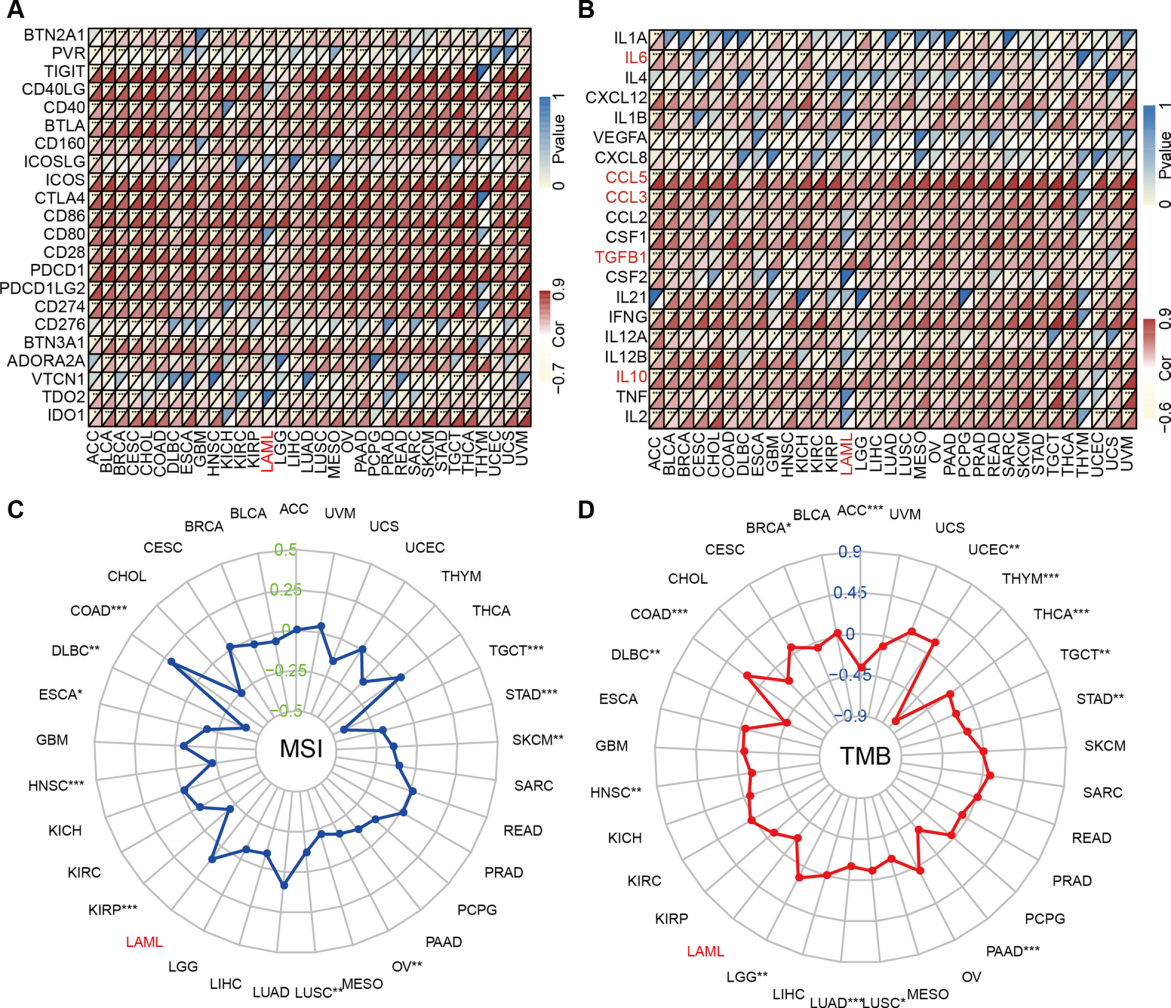
Heatmaps for the association of ITGAL with immune checkpoint genes (**A**), and cytokines (**B**)

We found that ITGAL was positively associated with most immune checkpoint genes and cytokines (Fig. 6A, B). There was no significant association between ITGAL and MSI and TMB in AML. However, a significant relationship was observed in other cancers, such as colon adenocarcinoma (Fig. 6C, D).

### Biological effects of ITGAL on human AML cells

**Fig. 7:**
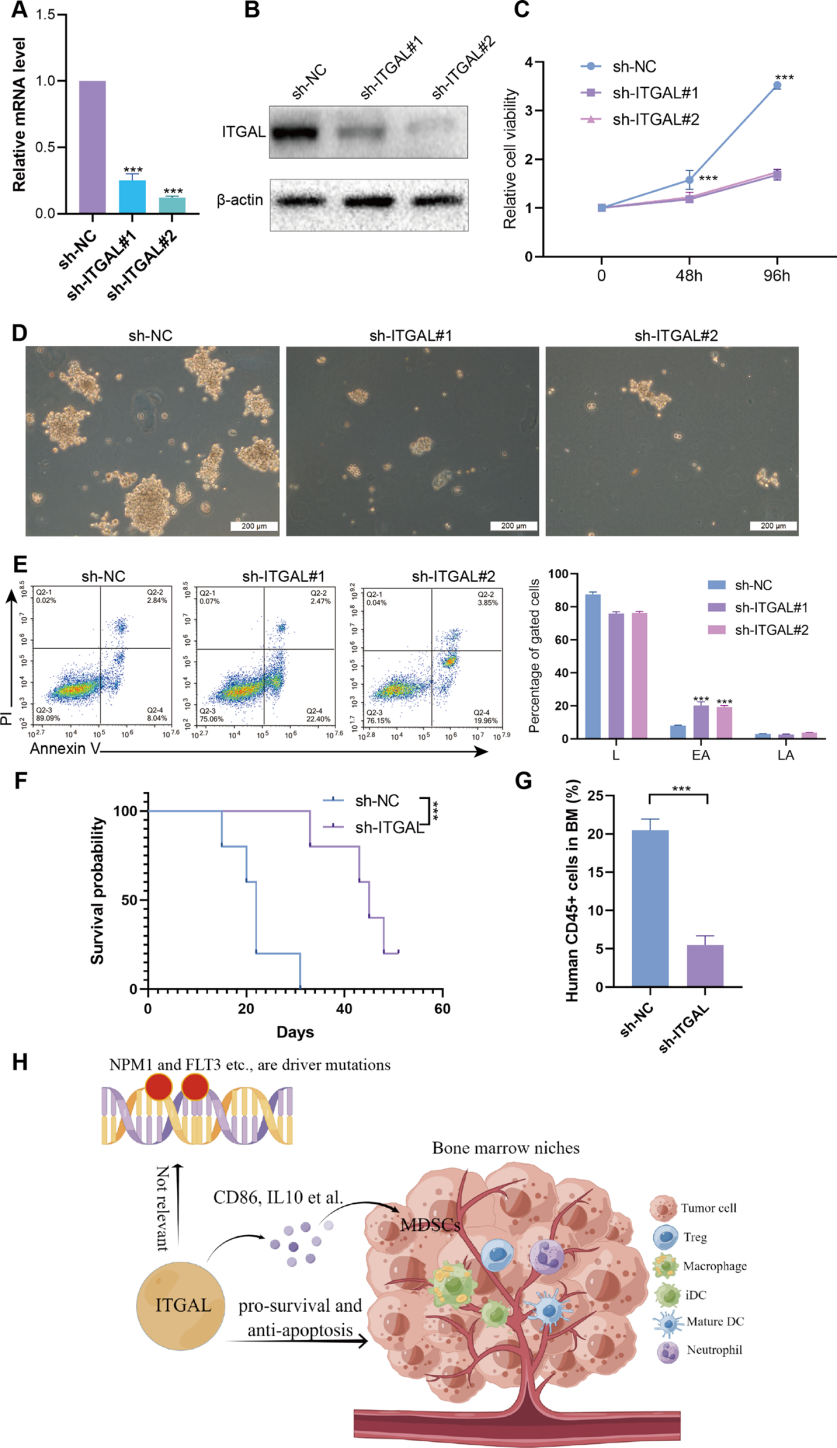
ITGAL promotes the growth of human AML cells. The mRNA (**A**) and protein (**B**) of ITGAL detection in MOLM13 cells transduced with sh-NC or sh-ITGAL. **C** Cell growth assays in MOLM13 cells. **D** Representative pictures of colonies from MOLM13 cells. **E** Representative flow cytometry plots of the percentage of apoptotic cells in MOLM13 cells with sh-NC or sh-ITGAL. L: living cells; EA: early apoptosis; LAL: late apoptosis. **F** Kaplan–Meier survival curves of NOD mice injected with MOLM13 cells transduced with sh-NC or sh-ITGAL. **G** The percentages of CD45+ cells in recipient mice. **H** The possible role of ITGAL in the AML progression, drawn by Figdraw. This diagram is largely based on pure bioinformatics analyses, which need to be validated using wet-lab experiments

For the final evaluation of ITGAL’s role in AML, it is necessary to perform tests based on their direct effect on AML cells. ITGAL knockdown cell model was established (Fig. 7A, B). Loss-of-function studies were performed and knockdown of ITGAL caused substantial inhibition of cell growth (Fig. 7C, D) and significant induction of early apoptosis in the MOLM13 cell line (Fig. 7E). In addition, ITGAL knockdown significantly prolonged survival in xenograft recipient mice (Fig. 7F). ITGAL knockdown also decreased CD45^+^ cells in BM and reduced spleen weight (Fig. 7G). Finally, the possible role of ITGAL in AML progression were shown in Fig. 7H. We found that ITGAL may affect both AML cells themselves and the immune tumor microenvironment to exert its oncogenic roles.

## Discussion

In the present study, a series of bioinformatics methods identified ITGAL as an adverse prognostic factor in AML. Functional analyses indicated that regulation of cytokine production may be the main mechanism of ITGAL-mediated AML progression. In addition, ITGAL may promote AML progression via regulating MDSCs.

Functional enrichment analysis showed that DEGs between high-ITGAL and low-ITGAL were most associated with cytokine production and cytokine metabolic process, indicating cytokine-related biological processes may play an important role in ITGAL-medicated AML progression. Through the co-expression analysis, we found that ITGAL was positively associated with IL-6, CCL5, CCL3, TGFB1, and IL-10 expression. Cytokines are potent regulators of a wide range of cellular functions and activities, especially in the immune system [19]. Consulting the literature revealed that elevated expression of these cytokines positively associated with ITGAL expression were all involved in the promotion of tumor cell survival, stemness, and proliferation [20–22]. Cytokines above exert their biological actions through diverse signal transduction pathways. For example, stimulating hyperactivation of JAK/STAT3 signaling [23] and activation of β-catenin/STAT3 signaling [24] corresponded to the role of IL-6 and CCL5, respectively.

In immune infiltration analysis, we found that ITGAL had a significantly positive correlation with iDC cells, macrophages, and Treg cells. iDCs and macrophages belong to the myeloid-derived suppressor cells (MDSC), which are a group of immature myeloid cell lineage cells [25]. These cells can suppress both innate and adaptive immune activities by suppressing mainly T cells, B cells, and NK cells [26]. In addition, the ability of MDSCs to promote the de novo development of Treg cells in vivo has been described [27]. Tregs are produced mostly by the thymus and are involved in maintaining immune tolerance as a functionally mature and distinct subpopulation of T cells [28]. The CD8^+^/FoxP3^+^ T cell ratio was commonly studied to predict the prognosis of patients and a meta-analysis indicated that a high CD8^+^/FoxP3^+^ T cell ratio was independently associated with improved survival based on the data from multiple cancer types [29]. MDSCs were associated with a worse response to cancer treatment and poor survival in patients with solid and hematological tumors [30]. We found that ITGAL was positively associated with MDSCs in AML patients. Hence, promoting the expansion of MDSCs may be another mechanism of ITGAL-mediated AML progression.

Among ten hub genes from the PPI analysis, CD86 and CD163 were biomarkers on the surface of M1- and M2-like macrophages respectively, and the high expression of CD86 and CD163 was related to the poor prognosis [31]. IL-10 secreted by M-MDSC plays a role in mediating tumor metastasis and suppressing immune response [32]. Human M-MDSC and G-MDSC produced CYBB/NOX2-derived ROS and inhibited T cell function [33, 34]. In addition, CCR5 promoted tumor development and progression by recruiting Treg and MDSC [35, 36]. These hub genes might play a vital role in the ITGAL-mediated expansion of MDSCs.

In conclusion, cytokines have been extensively investigated as either cancer targets or cancer treatments. This rationale is underscored by the discovery of altered and dysregulated cytokine expression in all human cancers. Malignantly transformed cells and AML progression lead to an immunosuppressed microenvironment, limiting the efficacy of various immunotherapies. The aberrant expansion of MDSCs is partially responsible for the formation of the immunosuppressed microenvironment in AML. We find that ITGAL is an independent prognostic factor and exerts a pro-oncogenic role, possibly through regulating cytokine production and the number of MDSCs. Hence, ITGAL may be a promising therapeutic target in AML patients.

## Supplementary Information

**Additional file 1: Table S1.** The characteristics of ITGAL in inclusive samples. **Table S2.** The univariate and multivariate analysis of clinical factors in AML samples. **Figure S1.** Kaplan–Meier survival curves for high- and low-ITGAL groups. Tertiles (A) or quartiles (B) of ITGAL expression were used as the cut-off value. **Figure S2.** A novel nomogram for AML prognosis. (A) Nomogram for predicting the probability of 1-, 3-, 5-year OS for AML. (B) Calibration plot of the nomogram. (C) ROC analysis for the nomogram.

## Data Availability

Data were obtained from the TCGA-LAML cohort (https://portal.gdc.cancer.gov/) and GEPIA (http://gepia.cancer-pku.cn/).
