# Supplementary material for: ITGAL infers adverse prognosis and correlates with immunity in acute myeloid leukemia
Source: Cancer Cell Int. 2022 Aug 23;22:268. doi: 10.1186/s12935-022-02684-x (PMC9400260; doi:10.1186/s12935-022-02684-x)
Supplement: Supplementary file 1 — Additional file 1: Table S1. The characteristics of ITGAL in inclusive samples. Table S2. The univariate and multivariate analysis of clinical factors in AML samples. Figure S1. Kaplan–Meier survival curves for high- and low-ITGAL groups. Tertiles (A) or quartiles (B) of ITGAL expression were used as the cut-off value. Figure S2. A novel nomogram for AML prognosis. (A) Nomogram for predicting the probability of 1-, 3-, 5-year OS for AML. (B) Calibration plot of the nomogram. (C) ROC analysis for the nomogram. [file 12935_2022_2684_MOESM1_ESM.docx]

Table S1. The characteristics of ITGAL in inclusive samples

| Characteristic | Low expression of ITGAL | High expression of ITGAL | p |
| --- | --- | --- | --- |
| n | 75 | 76 |  |
| Age, n (%) |  |  | 0.056 |
| <=60 | 50 (33.1%) | 38 (25.2%) |  |
| >60 | 25 (16.6%) | 38 (25.2%) |  |
| Gender, n (%) |  |  | 0.928 |
| Female | 33 (21.9%) | 35 (23.2%) |  |
| Male | 42 (27.8%) | 41 (27.2%) |  |
| OS event, n (%) |  |  | 0.023 |
| Alive | 34 (22.5%) | 20 (13.2%) |  |
| Dead | 41 (27.2%) | 56 (37.1%) |  |
| WBC count(x10^9/L), n (%) |  |  | 0.412 |
| <=20 | 41 (27.3%) | 36 (24%) |  |
| >20 | 33 (22%) | 40 (26.7%) |  |
| Cytogenetic risk, n (%) |  |  | 0.029 |
| Favorable | 21 (14.1%) | 10 (6.7%) |  |
| Intermediate | 33 (22.1%) | 49 (32.9%) |  |
| Poor | 19 (12.8%) | 17 (11.4%) |  |
| FAB classifications, n (%) |  |  | < 0.001 |
| M0 | 8 (5.3%) | 7 (4.7%) |  |
| M1 | 17 (11.3%) | 18 (12%) |  |
| M2 | 25 (16.7%) | 13 (8.7%) |  |
| M3 | 14 (9.3%) | 1 (0.7%) |  |
| M4 | 5 (3.3%) | 24 (16%) |  |
| M5 | 3 (2%) | 12 (8%) |  |
| M6 | 1 (0.7%) | 1 (0.7%) |  |
| M7 | 1 (0.7%) | 0 (0%) |  |
| FLT3 mutation, n (%) |  |  | 0.680 |
| Negative | 49 (33.3%) | 53 (36.1%) |  |
| Positive | 24 (16.3%) | 21 (14.3%) |  |
| NPM1 mutation, n (%) |  |  | 0.693 |
| Negative | 60 (40%) | 57 (38%) |  |
| Positive | 15 (10%) | 18 (12%) |  |
| Age, meidan (IQR) | 53 (39.5, 63.5) | 60.5 (45, 68.25) | 0.039 |

Table S2. The univariate and multivariate analysis of clinical factors in AML samples

| Characteristics | Total(N) | Univariate analysis | |  | Multivariate analysis | |
| --- | --- | --- | --- | --- | --- | --- |
|  |  | Hazard ratio (95% CI) | P value |  | Hazard ratio (95% CI) | P value |
| Age | 140 |  |  |  |  |  |
| <=60 | 79 | Reference |  |  |  |  |
| >60 | 61 | 3.333 (2.164-5.134) | **<0.001** |  | 2.696 (1.716-4.235) | **<0.001** |
| WBC count(x10^9/L) | 139 |  |  |  |  |  |
| <=20 | 75 | Reference |  |  |  |  |
| >20 | 64 | 1.161 (0.760-1.772) | 0.490 |  |  |  |
| Cytogenetic risk | 138 |  |  |  |  |  |
| Favorable | 31 | Reference |  |  |  |  |
| Poor&Intermediate | 107 | 3.209 (1.650-6.242) | **<0.001** |  | 2.126 (1.063-4.252) | **0.033** |
| ITGAL | 140 | 1.394 (1.154-1.684) | **<0.001** |  | 1.275 (1.028-1.582) | **0.027** |


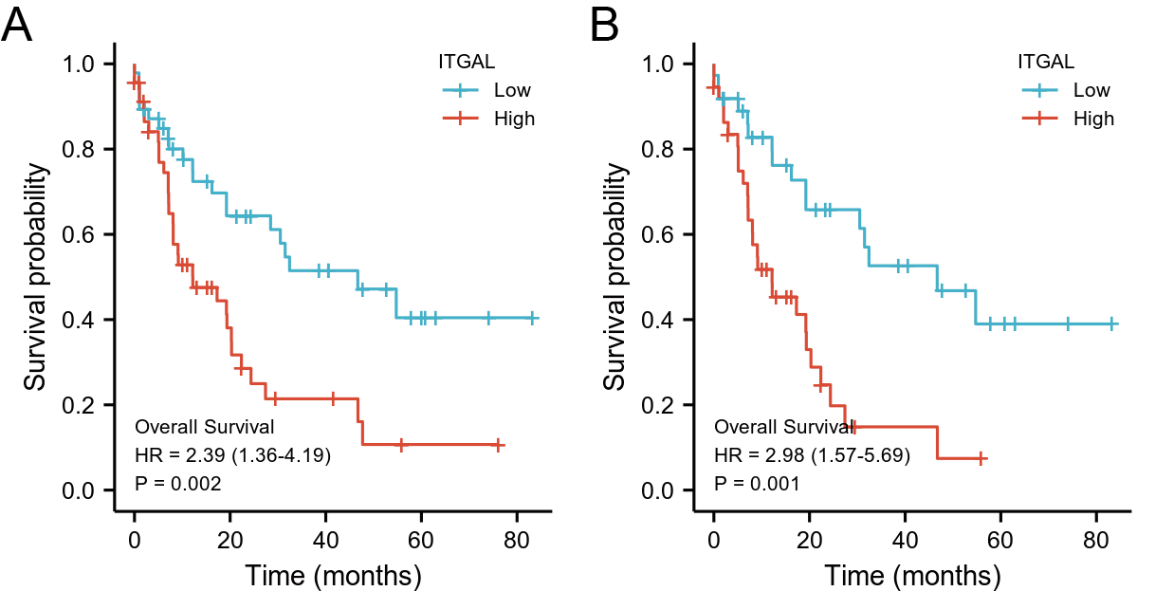


**Figure S1. Kaplan-Meier survival curves for high- and low-ITGAL groups.** Tertiles (A) or quartiles (B) of ITGAL expression were used as the cut-off value.


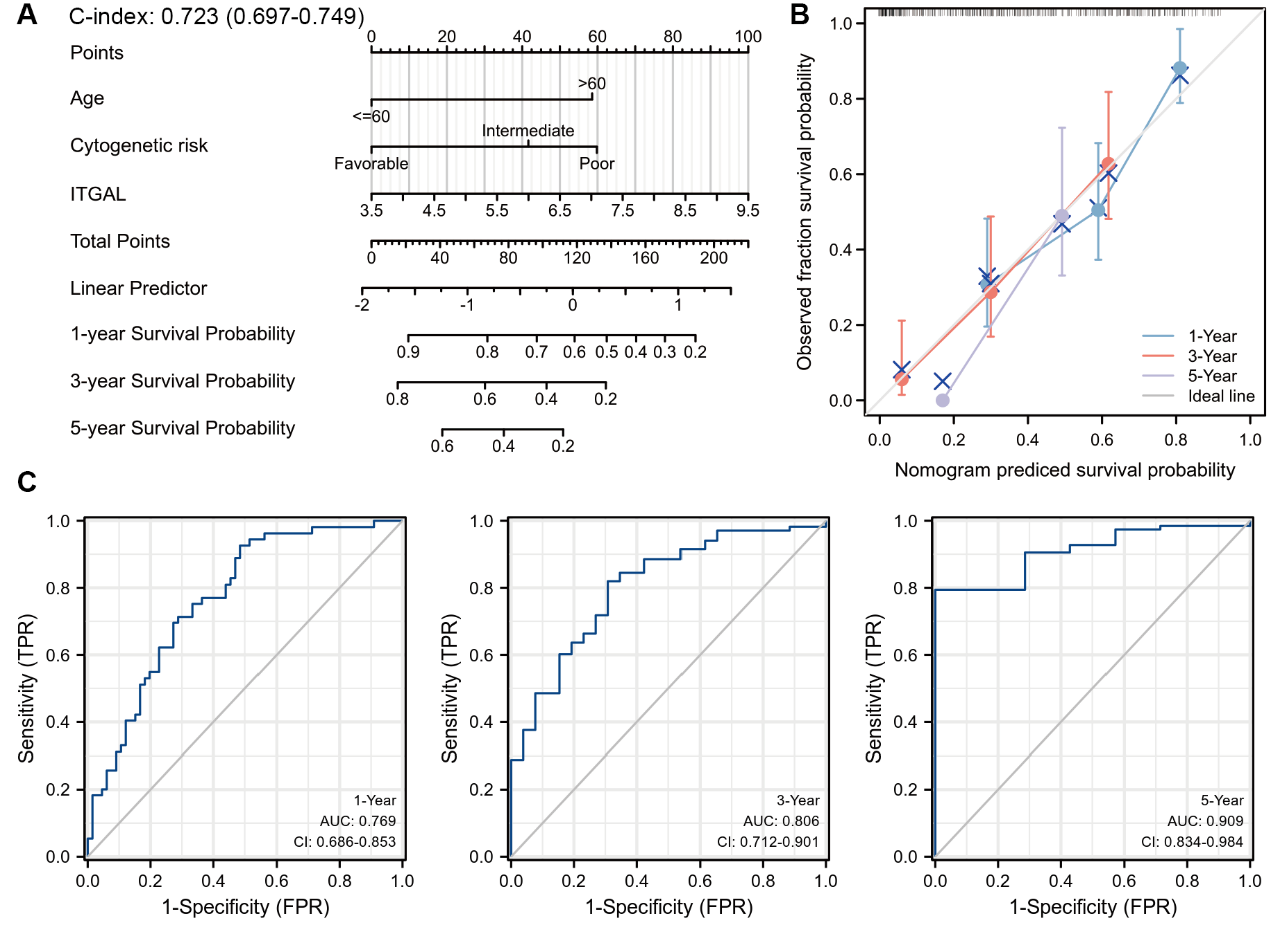


**Figure S2. A novel nomogram for AML prognosis.** (A) Nomogram for predicting the probability of 1-, 3-, 5-year OS for AML. (B) Calibration plot of the nomogram. (C) ROC analysis for the nomogram.
